# Evanescent wave quartz-enhanced photoacoustic spectroscopy employing a side-polished fiber for methane sensing

**DOI:** 10.1016/j.pacs.2024.100586

**Published:** 2024-01-22

**Authors:** Cian F. Twomey, Gabriele Biagi, Albert A. Ruth, Marilena Giglio, Vincenzo Spagnolo, Liam O’Faolain, Anton J. Walsh

**Affiliations:** a Centre for Advanced Photonics and Process Analysis, Munster Technological University, Cork, T12 P928, Ireland; b PolySense Lab, Dipartimento Interateneo di Fisica, University and Politecnico of Bari, Bari, CNR-IFN, Via Amendola 173, Bari 70126, Italy; c School of Physics and Environmental Research Institute, University College Cork, Cork, Ireland; d Tyndall National Institute, Lee Maltings Complex Dyke Parade, Cork, T12 R5CP, Ireland

**Keywords:** Quartz-enhanced photoacoustic spectroscopy (QEPAS), Evanescent wave QEPAS, Side-polished fiber, Methane leak detection, Parts-per-million sensitivity, Gas detection, Evanescent wave spectroscopy

## Abstract

We present an all-fiber-based laser gas analyzer (LGA) employing quartz-enhanced photoacoustic spectroscopy (QEPAS) and a side-polished fiber (SPF). The LGA comprises a custom quartz tuning fork (QTF) with 0.8 mm prong spacing, two acoustic micro-resonators (mR) located on either side of the prong spacing, and a single-mode fiber containing a 17 mm polished section passing through both mRs and QTF. The SPF polished face is positioned to enable the evanescent wave (EW) to create a photoacoustic wave and excite the fundamental flexural mode of the QTF. Sensor performance was demonstrated using methane in nitrogen gas mixtures, with CH${}_{4}$ mixing ratios ranging from 75 ppmv to 1% (by volume), measured with an accumulation time of 300 ms, and a minimum detection limit of 34 ppmv subsequently determined. The EW-QEPAS sensor is ideal for miniaturization, as it does not contain any free-space optics and is suitable for gas sensing in harsh environments and where mobility is required.

## Highlights

• First demonstration of evanescent wave QEPAS using a side-polished fiber.
• All-fiber-based QEPAS with no free-space optics used for methane sensing.
• Parts-per-million sensitivity for methane in the near-infrared region using EW-QEPAS.

## Graphical abstract

**Figure:**
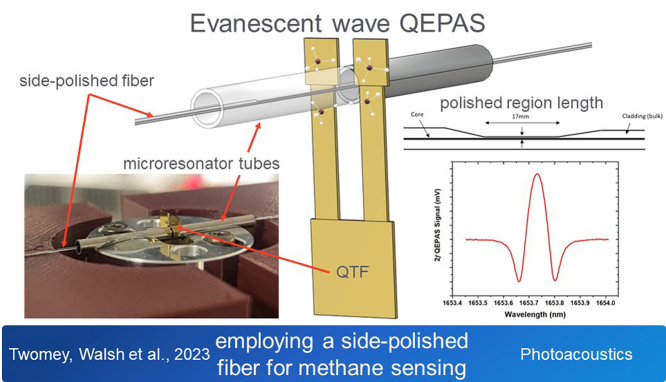

## 1 Introduction

Laser gas sensing technology has developed rapidly in recent years due to the availability and progress in solid-state and semiconductor laser technology. The introduction of Distributed Feedback (DFB) laser diodes, DFB-Interband Cascade Lasers (DFB-ICLs), and DFB-Quantum Cascade Lasers (DFB-QCLs) has revolutionized the field of gas sensing and spectroscopy [1]. The Laser Gas Analyser (LGA) market includes detectors based on a variety of spectroscopic techniques, including photoacoustic, tunable diode laser absorption, off-axis integrated cavity output, cavity ringdown, and cavity enhanced phase shift spectroscopy [2]. LGA detectors benefit from being non-destructive, while still offering high selectivity, time resolution, and precision. For example, methane mixing ratios with a precision of 0.025 ppbv (in 100 s) and 0.5 ppbv (in 1 s) have been measured using commercial laser-based instruments [3]. However, a limitation of most LGA detectors is their free-space optics configuration and low misalignment tolerance, i.e., the relative position of mirrors and lens along the beam path, a crucial parameter for high sensitivity and sensor lifetime. Consequently, the performance of LGAs is generally challenged by harsher environments, where mechanical vibrations can occur, e.g., in agricultural or industrial settings, and where portability is crucial. The development of more robust sensors through the containment of light, e.g., in optical fibers or silicon photonic waveguides demonstrates substantial potential. Employing evanescent waves (EW) to interact with the sample analyte is a promising approach to circumvent the need for sensitive alignment of free space optics. The electric field of the EW decays exponentially with distance from the interface at which they are formed. Confining light within fibers or waveguides can also reduce the LGA footprint and integration complexity [4]. For optical fibers, side polishing [5] and flame tapering [6] are common techniques used to provide analyte access to the EW. In a direction along the fiber pathlengths on the order of a few centimeters are typically achieved. Such pathlengths do not provide good sensitivity using direct absorption spectroscopy. However, short pathlengths are applicable to photoacoustic spectroscopy, such as quartz-enhanced photoacoustic spectroscopy [7], [8].

Methane (CH${}_{4}$) is one of the most important greenhouse gases, second to CO${}_{2}$, with a global mean concentration of ≈ 1.9 ppmv in the atmosphere in 2021 [9], it is a major contributor to climate change, with a global warming potential of 84–87 times that of CO${}_{2}$ over 20 years. Methane plays an important role across multiple industries, including oil/gas mining, agriculture, and health. Methane is flammable and explosive above 4% concentrations in air and monitoring of methane, at relatively high mixing ratios of a few percent, is required in petrochemical industries to satisfy health and safety requirements [10], [11]. The Occupational Safety and Health Administration (OSHA) in the United States, set the permissible exposure limits for methane at 1000 ppmv over an 8-hour time-weighted average. Consequently, mobile and resilient sensors, suitable for the majority of work environments, with sub-100 ppmv sensitivity are desirable. Agriculture is the largest source of anthropogenic methane emissions worldwide, accounting for 43.6% of global emissions [12].

In the livestock sector, up to 81% of methane emission is from animal breath, of which 90% is due to the methanogenesis of microbes in the stomachs of ruminant animals [13]. Dairy cows account for about 15% of the global methane budget, where several hundred ppmv of methane (typically 600–1200 ppmv), have been measured in their breath [14]. Similar to leak detection in the oil/gas industry, sub-100 ppmv sensitivity is not always needed, but mobility and sensor robustness are required. Methane is an important biomarker in medicine whose mixing ratios in human breath, are usually very low, in the region of only 2–10 ppmv [15]. However, when present excessively in human breath it can indicate intestinal or colon-related diseases, oxidative stress, breast cancer, hepatic disease, heart disease, obesity, and anorexia [15], [16]. In analogy to dairy breath analysis and workplace leak detection, high sensitivity is not required, and compactness, mobility, and robustness are pertinent. This study presents an all-fiber-based laser gas analyzer that can meet the criteria outlined above while offering a sensitivity of 34 ppmv in an integration time of 300 ms. This was demonstrated using methane in nitrogen gas mixtures, with CH${}_{4}$ mixing ratios ranging from 75 ppmv to 1% (by volume).

### 1.1 Evanescent wave QEPAS

QEPAS employs a quartz tuning fork (QTF) to detect photoacoustic waves generated by the non-radiative energy relaxation of the target gas molecules excited by a modulated laser source. In the second harmonic (2*f*) detection scheme, the probing light is wavelength modulated at half the fundamental in-plane flexural mode frequency ($f_{\text{QTF}}$) of the QTF and focused between the two prongs of the QTF, which consequently vibrate along opposite directions within the QTF plane [8]. The mechanical bending of the quartz prongs resonates with the acoustic waves and generates an electrical signal by the direct piezoelectric effect. The piezo-current is amplified using a transimpedance amplifier and subsequently, the second harmonic is demodulated by a lock-in amplifier. The demodulated signal is proportional to (i) the target gas mixing ratio, (ii) the exciting optical power, (iii) the QTF quality factor, and (iv) the radiation-to-sound conversion efficiency. QEPAS does not require any optical detector and has good immunity to environmental noise. In most cases, micro-resonator tubes (mR) are acoustically coupled to the QTF to generate standing waves and enhance the acoustic waves amplitude, improving the QEPAS signal-to-noise ratio up to a few tens of times [17]. The laser beam, which enters the mR, must not hit the inner wall surface or the QTF prongs to avoid photothermal effects, which is one of the main sources of noise in PAS. Laser fluctuations will add noise to the sensor signal. Consequently, it is recommended to place an optical power meter at the light exit of the spectrophone to account for variations. QEPAS has been successfully applied from the UV–visible to the THz range, using LED, semiconductor laser, ICL, and QCL sources [8], [18].

Optical fiber components have been introduced in QEPAS as a replacement for free-space bulk optics, such as lenses, polarizers, and mirrors to improve compactness and sensor network/multipoint-detection compatibility, while at the same time reducing interference artifacts and the risks of misaligning the optical components [19], [20]. The majority of fiber optic QEPAS configurations, however, remain susceptible to misalignment, as free-space optics are required to direct the beam from the fiber output to the spectrophone. To circumvent the need for any free-space optics, EW approaches have been developed, where tapered optical fibers are placed between the QTF prongs to enable the exposure of the gas sample to the EW [4], [6], [21]. Typically, fibers are tapered by reducing the cladding thickness to a few μm using a flame, with sensitivity increasing with reduced cladding thickness. A Minimum Detection Limit (MDL) of 20 ppmv at an averaging time of 210 s was demonstrated for carbon monoxide using tapered fiber EW-QEPAS [21]. In addition, EW-QEPAS allows for staggered detection, where multiple points on the fiber are tapered. Mixing ratios of acetylene, as low as 13 ppmv, have been measured at 3 tapered points on a single 3 km fiber [4].

Applications of tapered fibers to optical sensors have their challenges [22]. Manufacturing of tapered fibers has limited reproducibility, resulting in limited dependable sensitivity for EW-QEPAS [4]. Tapered fibers cannot be produced in bulk, restricting possible production yields. Finally, there are difficulties due to weak mechanical strength and delicate handling. An alternative approach is to use side-polished fibers, where access to the EW field is generated through grinding and polishing the cladding on one side.

### 1.2 Side-polished fibers and their applications

Side-polished fibers (SPF) have a portion of the fiber cladding removed by polishing, resulting in a D-shaped cross-section in the polished region. SPFs have been generated from single-mode, polarization maintaining, multi-mode, and photonic crystal fibers [5]. Two methods are commonly used to fabricate SPFs: (a) the *V-groove assisted polishing technique* [23], where the fiber is supported in V-grooves and is ground and polished simultaneously, and (b) the *wheel polishing technique* [24], where the fiber is suspended tightly below a motorized grinding wheel coated uniformly with a combination of abrasive paper and lubricant oil. The *wheel polishing technique* is flexible in tailoring polished length and depth, has high reproducibility, is low-cost, and allows multiple fibers to be polished simultaneously. Subsequently, all-fiber-based devices employing SPFs offer commercial opportunities, compared to other evanescent field-based tapered fiber systems, due to the potential large-scale fabrication and low-cost *wheel polishing techniques* [24], [25]. The SPF used in this paper was commercially sourced.

SPFs have been used in refractive index sensors [26], humidity sensors [27], temperature sensors [28], UV sensors [29], acoustic pressure sensors [30] and strain sensors [31]. Outside of sensing, SPFs have also been successfully applied for all-fiber devices, e.g., filters, couplers, optical resonators, polarizers, and attenuators [5].

## 2 Material and methods

The SPF acquired from Phoenix Photonics (SPF-S-SM-2) was made from a standard SMF28 optical fiber, with core and cladding diameters of 8.2 and 128μm respectively. The SPF contains a 17 mm polished section, made using the wheel polishing technique, and is optimized for use between 1525 and 1625 nm. The depth to which the cladding is removed from the fiber gives control over the level of interaction between the sample and the evanescent field. The SPF was cleaved on one side, to allow the polished section of the fiber to pass through the acoustic resonators and QTF of the QEPAS acoustic detection module (ADM), and was connected to an optical fiber vacuum feedthrough using an FC/APC connector. The side-polished surface was always facing up, away from the base of the tuning fork. The same position of the polished section face was kept during measurement, which was found empirically by rotating the fiber and remeasuring it under the same conditions.

The laser source used was a Discrete Mode fiber pig-tailed laser diode from Eblana Photonics (EP1654-DM-B), lasing at 1653.7 nm (6047 cm^−1^) where a C-H stretch absorption overtone of methane is located [32]. The laser was characterized as a function of temperature and current using a Laser Diode Controller (Stanford Research Systems LDC501) and an Optical Spectrum Analyzer (Ando AQ6317B), see supplementary material Figure S1. While keeping the temperature constant, the current driving the laser was modulated with a 10 mA ramp center at 88.85 mA, enough to sweep the laser wavelength over the entire methane absorption feature. The peak-to-peak current modulation depth was 5.1 mA. Part of the measurements have been performed by implementing a Booster Optical Amplifier (BOA) (Thorlabs BOA1550P) capable of increasing the laser power from 6.7 to 80 mW. The BOA was kept at a constant temperature of 25 °C and a constant current of 600 mA (Thorlabs Laser Diode Controller CLD1015).

**Fig. 1:**
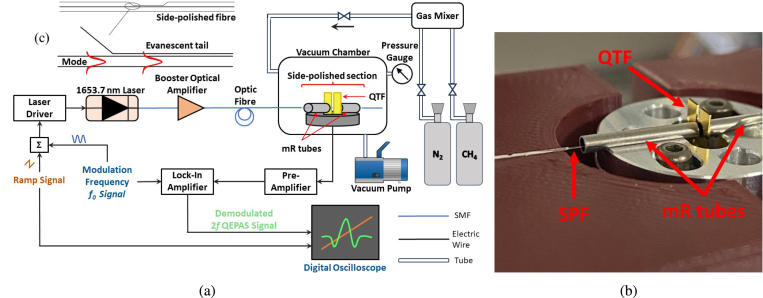
(a) Schematic of EW-QEPAS setup, (b) Image of spectrophone and SPF mount, (c) schematic of an SPF and side-polished region schematically showing mode propagation and access to the evanescent tail of the mode. All light coupling from the laser to the side-polished fiber was done using optical fibers.

A schematic of the EW-QEPAS system is shown in Fig. 1(a), with an image of the spectrophone/SPF alignment shown in Fig. 1(b). A schematic showing the side-polished region granting access to the evanescent tail of the propagating mode in fiber is shown in Fig. 1c. To overcome the limitations of standard QTFs, a custom QTF, labeled S08, and a commercial QEPAS acoustic detection module (Thorlabs ADM01) were used in this work. The frequency of laser modulation, corresponding to the resonance frequency of the QTFs, should be selected to be lower than the effective relaxation rate of the analyte within the gas matrix, to facilitate efficient acoustic signal generation [17]. The S08 model of QTF, characterized by a prong spacing of 0.8 mm, exhibits a fundamental resonance frequency of 12434.9 Hz and a quality factor of >12000 under atmospheric pressure conditions. This frequency is particularly advantageous for analytes with slower relaxation dynamics, such as methane, as evidenced in the research conducted by [33], [34], particularly in the context of wet mixtures. It was coupled with millimeter-sized resonator (mR) tubes with an inner diameter of 1.6 mm in a dual-tube on-beam configuration, i.e., with the laser beam passing through the mR-tubes and between the QTF prongs.

The commercial ADM needed to be adapted to employ the SPF. Two custom fiber holders were designed to be attached to the ADM. The fiber holders had a V-groove to keep fiber alignment in place, with the polished section located between the QTF prongs and inside the mR tubes. The unpolished section was secured to the fiber holder with a reusable adhesive. The fiber was placed on the beam optical axis used in free-space QEPAS to excite the first harmonic frequency, with the polished region facing out from the base of the QTF. The response of the QTF during module movement and chamber evacuation/filling was monitored by applying a voltage modulation to the QTF at its resonance frequency $f_{\text{QTF}}$. This ensured that any misalignment of the fiber, causing the fiber to touch the QTF, could be noticed immediately and corrected.

Between measurements, the chamber was evacuated to below 2 mbar using a vacuum pump and purged using pure nitrogen gas (Nitrogen CP Grade N5.2) for 5 min to remove any residual air or gases from the chamber. The nitrogen was then slowly vented from the chamber by closing the nitrogen supply valve and evacuating the chamber. The chamber was then filled at various methane mixing ratios using a gas mixer (MCQ GB100), a cylinder of 500 ppmv or 1% methane (by volume) in nitrogen, and a cylinder of pure nitrogen gas.

During QEPAS measurements, the laser current was driven by the combination of a positive ramp, with a period of 60 s (Rigol DG5072), and a sine wave, with a frequency of half the QTF resonance $f_{0}$ = 6217.15 Hz (TTI TG1010), generating a photoacoustic wave of frequency 2$f_{0}$. The ADM contains a pre-amplifier to amplify the piezo-electric signal generated by the photoacoustic wave. The amplified signal was then demodulated at 2$f_{0}$ by a digital lock-in amplifier (Anfatec, USB Lock-in Amplifier 250).

## 3 Results

**Fig. 2:**
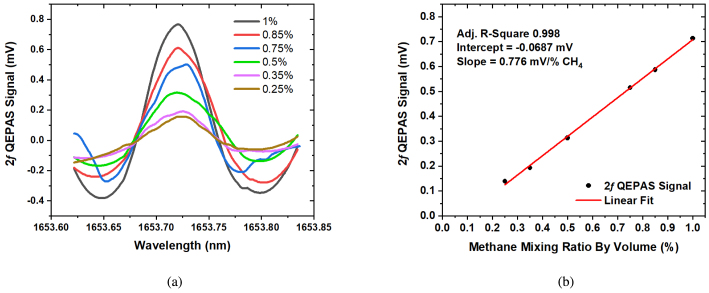
(a) Spectra of 2*f* QEPAS signals at methane mixing ratios between 0.25% and 1% in nitrogen gas, (b) Linear fit of 2*f* amplitudes as a function of methane mixing ratio, for mixing ratios between 0.25% and 1%. Collected using a digital lock-in amplifier with a time constant of 100 ms, a positive ramp with a period of 60 s covering 10 mA, and a modulation frequency of 6217.15 Hz with a depth of 5.1 mA. The 2*f* QEPAS Signal values in Fig. 3(b) were obtained by averaging over 4 points at the peak of each spectrum. The standard error of the intercept and slope was $9.98\times 10^{-6}$ mV/%CH${}_{4}$ and $1.48\times 10^{-5}$ mV, respectively.

### 3.1 Pressure optimization

A pressure optimization study was performed using a 500 ppmv mixture of methane diluted in nitrogen gas and employing the BOA to amplify laser power. The pressure was increased in steps of 200 mbar while recording the maximum demodulated QEPAS signal, varying modulation frequency and depth as the QTF resonance frequency is pressure dependent, see supplementary material Figure S2. The optimum pressure was determined to be ≈ 800 mbar. All measurements were performed at 800 mbar pressure, *f_0_* = 6217.15 Hz, current modulation depth of 5.1 mA, and 10 mA ramp signal.

### 3.2 EW-QEPAS with DFB laser as sole excitation source

The EW-QEPAS set-up was tested without the BOA amplification, using higher methane mixing ratios, up to 1% methane (by volume) diluted in nitrogen, see Fig. 2(a) for 2*f*${}_{0}$ demodulated spectra at different mixing ratios. The 1σ noise level was evaluated based on the standard deviation of measurement data when pure nitrogen gas filled the system and was found to be 0.071 mV, see supplementary material Figure S3. Using only the discrete mode semiconductor laser of ≈ 6.7 mW power, methane mixing ratios down to 0.099% can be detected with EW-QEPAS. Fig. 2(b) displays a linear regression of QEPAS response vs methane mixing ratio. The corresponding R-square value is 0.997. The linear function is described by a slope of 0.772 mV/% CH${}_{4}$, and an intercept of −0.065 mV. This intercept is within one standard deviation of the noise. This linearity and sensitivity of 0.25% are sufficient to monitor methane within the flammability/combustion regime.

### 3.3 Calibration, sensitivity, and limit of detection

**Fig. 3:**
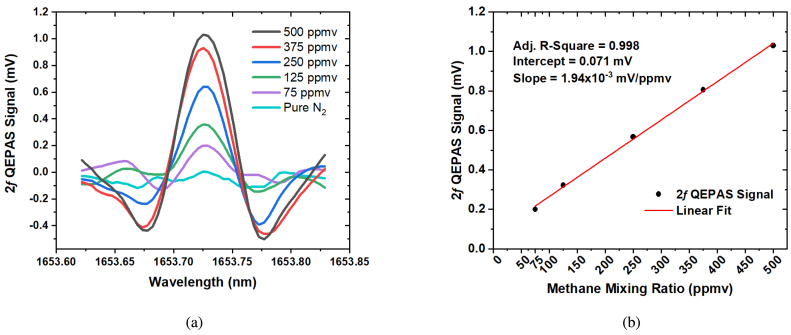
(a) Spectra of 2*f* QEPAS signals at methane mixing ratios between 75 and 500 ppmv in N${}_{2}$, (b) 2*f* amplitudes as a function of methane mixing ratio. The linear dependence for mixing ratios between 75 and 500 ppmv was described by a linear regression. Data was collected using a digital lock-in amplifier with a time constant of 100 ms, a positive ramp with a period of 60 s covering 10 mA, and a modulation frequency of 6217.15 Hz with a depth of 5.1 mA. The 2*f* QEPAS Signal values in Fig. 3(b) were obtained by averaging over 4 points at the peak of each spectrum. The standard error of the intercept and slope was $1.29\times 10^{-5}$ mV/ppmV and $4.18\times 10^{-8}$ mV, respectively. A different SPF than the one used for the measurements shown in Fig. 2 was used to collect this data.

The set-up sensitivity was increased significantly by the addition of the BOA optical amplifier between the SPF and the laser. Fig. 3(a) shows 2*f*${}_{0}$ demodulated QEPAS spectra at different methane mixing ratios in nitrogen between 500 and 75 ppmv measured with a different SPF.

A linear fit of QEPAS signal versus mixing ratio is shown in Fig. 3(b), with an R-square value of 0.998, a slope of $1.94\times 10^{-3}$ mV/ppmv, and an intercept of 0.071 mV, matching the measured noise level. The linearity of the sensor responsivity with the methane mixing ratio confirmed the reliable performance of the setup. An MDL of 34 ppmv CH${}_{4}$ at a lock-in time constant of 100 ms was calculated using the measured signal-to-noise ratio for the 500 ppm methane gas mixture.

## 4 Theory/calculations

**Fig. 4:**
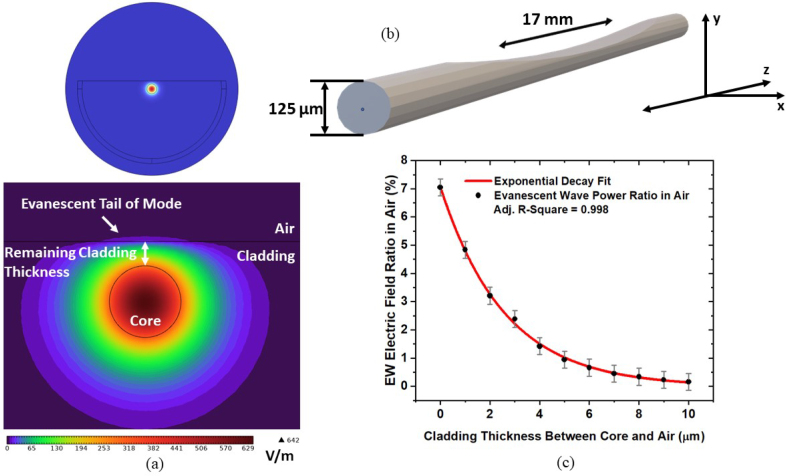
(a) Mode profiles of the full side-polished fiber at the center of the side-polished region in air (top) and near the surface of the side-polished region with 2.78μm remaining cladding thickness (below), showing the evanescent tail of the mode, recolored to facilitate visualization, (b) 3D model of a side-polished fiber showing the directions of axes used and the fiber dimensions, (c) exponential fit of evanescent wave power percentage in the air for increasing cladding thickness obtained from the COMSOL simulation.

To investigate the field distribution of the EW in air and the percentage of the EW in air a COMSOL Multiphysics 6.1 finite element method (FEM) simulation was employed. In the air environment, the EW remains near the surface of the polished section directly above the core and does not spread out along the width of the polished region, shown in Fig. 4a. These simulations show that the EW electric field is highest closer to the side-polished region surface, see Fig. 4a. The electric field of the EW at the surface and of the mode in the core of the fiber was obtained using a Mode Analysis. The corresponding results are shown in Fig. 4a together with a 3D model of the SPF in Fig. 4b, showing the dimensions of the SPF. The ratio of the EW electric field at the surface and the electric field in the core is used to determine the EW percentage in air, see Fig. 4c, as a function of the remaining cladding thickness. As the remaining cladding thickness increases the EW electric field, in the air at the surface it decreases exponentially.

An SPF was cleaved at the middle of the polished region to obtain an image of its cross-section using a Scanning Electron Microscope (SEM) (Hitachi S-3700N) and the cladding thickness obtained from the SEM image was 2.78μm. Based on the fit from Fig. 4c, 2.36% of the mode interacts with the methane for this thickness. According to the analysis, the 17 mm polished section should be placed close to the beam height optical axis used in free-space configurations, as the EW remains close to the surface of the fiber above the core. The midpoint of the polished region should be positioned directly between the QTF prongs as the cladding thickness is minimal at this point.

## 5 Conclusion

The first demonstration of a side-polished fiber for photoacoustic sensing has been made in an EW-QEPAS sensor. Both the SPF and QEPAS detection module for the constructed sensor were commercially sourced, highlighting that the fundamental components of the detector can be produced in quantities to make it commercially viable. Two configurations of the all-fiber-based sensor were presented for methane detection. The first employed a discrete mode pigtailed diode laser with a central wavelength of 1653.7 nm to generate the photoacoustic wave. This configuration allowed the detection of methane buffered in nitrogen at 0.25%, with a linear response in QEPAS signal as a function of mixing ratio. The sensor’s sensitivity is sufficient to monitor methane at flammable mixing ratio levels. A second configuration of the sensor, employing a BOA between the laser and the SPF, significantly increased its sensitivity. A 1σ detection limit of 34 ppmv for a 100 ms lock-in time constant was determined using the measured signal-to-noise ratio for the 500 ppmv methane gas mixture. A linear relationship between the EW-QEPAS signal and methane mixing ratio was obtained (R${}^{2}$ = 0.998). The EW-QEPAS sensor configuration including the BOA demonstrates the required sensitivity and linearity for many applications, including industrial leak detection [10], [11], dairy cow breath analysis [14], and has potential for monitoring excessive methane production in human breath [15].

While the MDL of the present set-up does not match other methane sensors, the setup is all fiber-based and hence has favorable features in terms of robustness, mobility, staggered detection capacity, and compactness compared to other sensors. As the difficulties of free space alignment are not present, the SPF can be easily employed with narrower prong spacings, which may improve sensitivity further. Bringing the QTF prongs closer to the SPF can increase the QEPAS signal. Different types of resonator configurations can be experimented with as the acoustic resonator diameters are no longer limited by the beam spatial profile, similar to reducing QTF prong spacing. The principal reason for the reduction in sensitivity, compared to free-space QEPAS, is the weaker excitation optical power. Adding coating or structures to the polished fiber, to extend the evanescent field and increase the EW electric field ratio [27], [35], will increase the excitation optical power of the SPF EW-QEPAS and consequently, increase sensitivity. The robustness of the SPF, compared to tapered fiber, allows this extension. As for all LGA sensors, the larger absorption cross-sections of fundamental vibrations in the mid-IR offer the possibility of higher sensitivity. Typical Interband- and Quantum Cascade Lasers (ICLs, QCLs) available for gas sensing in the MIR offer higher power than laser diodes available in the shortwave [36] and optic fiber coupling with single mode output of > 88% efficiency has been measured in the 3.7–7.6μm range [37]. Other EW approaches using Mid-IR single-mode fibers have been reported, including the measurement of real-time bioprocesses [38], fruit spoilage based on volatile compounds [39], organic constituents in marine environments [40], and in vitro glucose measurements [41]. EW sensing has also been reported using MIR SPFs, for measurement of glycerin concentrations in water [42] and the detection of the volatile organic compound butane [43] and their employment in EW-QEPAS can offer further increases in sensitivity. Consequently, several novel adaptations are viable to improve sensitivity further and expand possible real-world sensing applications.

## CRediT authorship contribution statement

**Cian F. Twomey:** Writing – review & editing, Writing – original draft, Investigation, Formal analysis, Data curation, Conceptualization. **Gabriele Biagi:** Writing – review & editing, Investigation, Formal analysis, Data curation, Conceptualization. **Albert A. Ruth:** Writing – review & editing, Resources, Investigation. **Marilena Giglio:** Writing – review & editing, Investigation, Formal analysis, Conceptualization. **Vincenzo Spagnolo:** Writing – review & editing, Investigation, Funding acquisition, Formal analysis, Conceptualization. **Liam O’Faolain:** Writing – review & editing, Resources, Investigation, Funding acquisition, Formal analysis, Conceptualization. **Anton J. Walsh:** Writing – review & editing, Investigation, Funding acquisition, Formal analysis, Data curation, Conceptualization.

## Declaration of competing interest

The authors declare that they have no known competing financial interests or personal relationships that could have appeared to influence the work reported in this paper.

## Data Availability

Data will be made available on request.

## Appendix A Supplementary data

MMC S1 .

MMC S2 .

MMC S3 .
