# Supplementary figures and images for: Evanescent wave quartz-enhanced photoacoustic spectroscopy employing a side-polished fiber for methane sensing
Source: Photoacoustics. 2024 Jan 22;36:100586. doi: 10.1016/j.pacs.2024.100586 (PMC11636781; doi:10.1016/j.pacs.2024.100586)

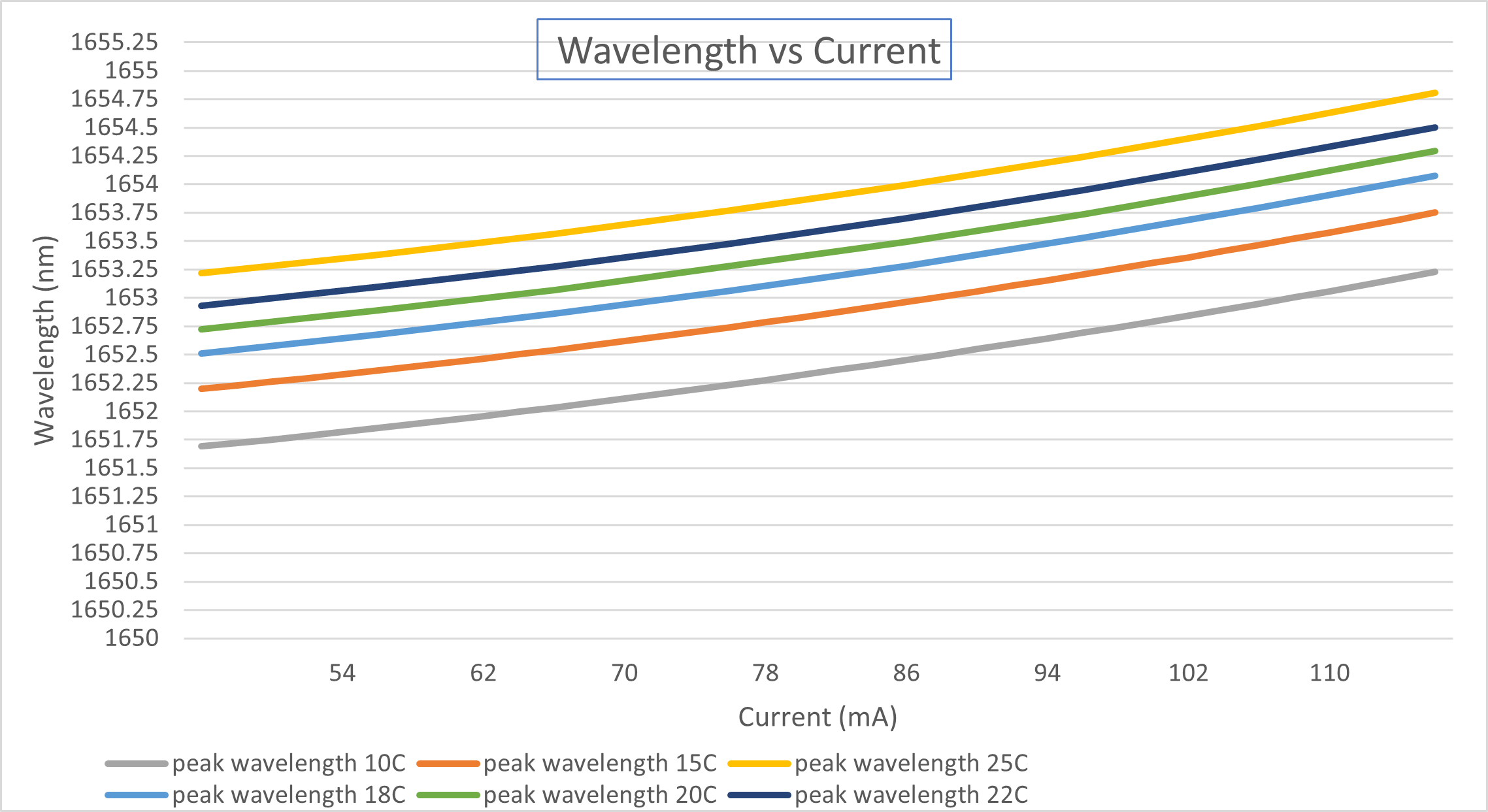

Supplement: MMC S1 — . [file mmc1.zip › Figure_S1_V2.png]

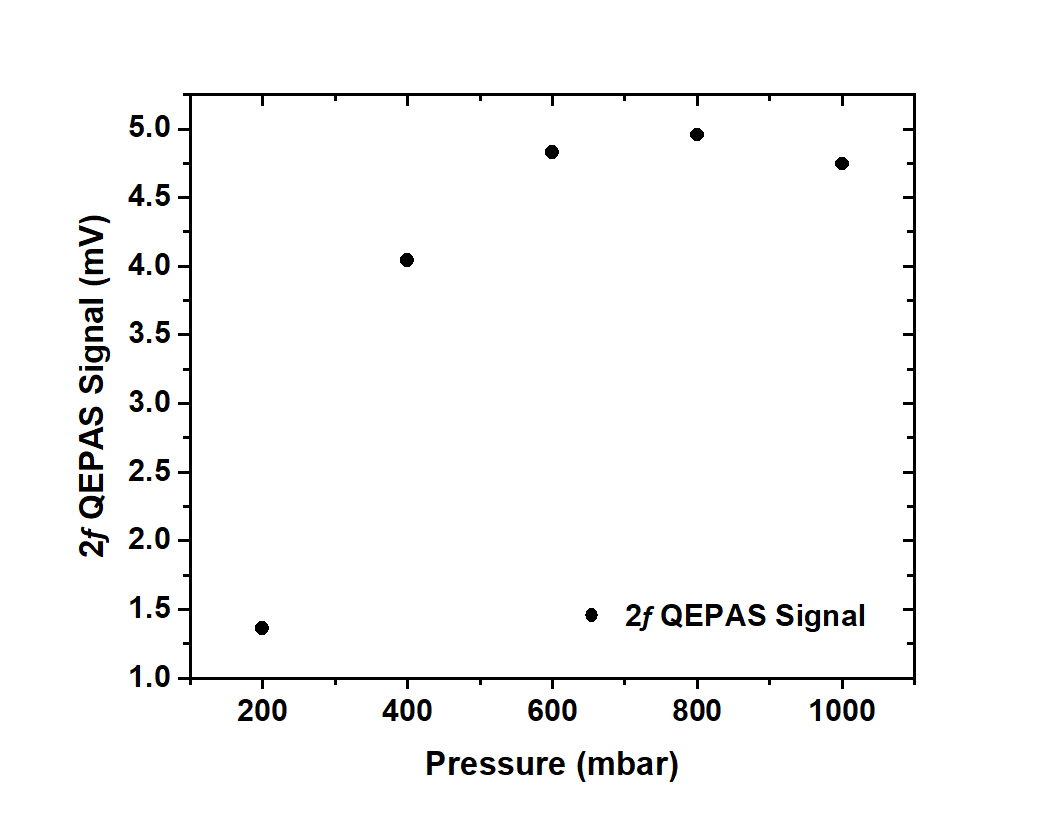

Supplement: MMC S2 — . [file mmc2.zip › Figure_S2_V2.jpg]

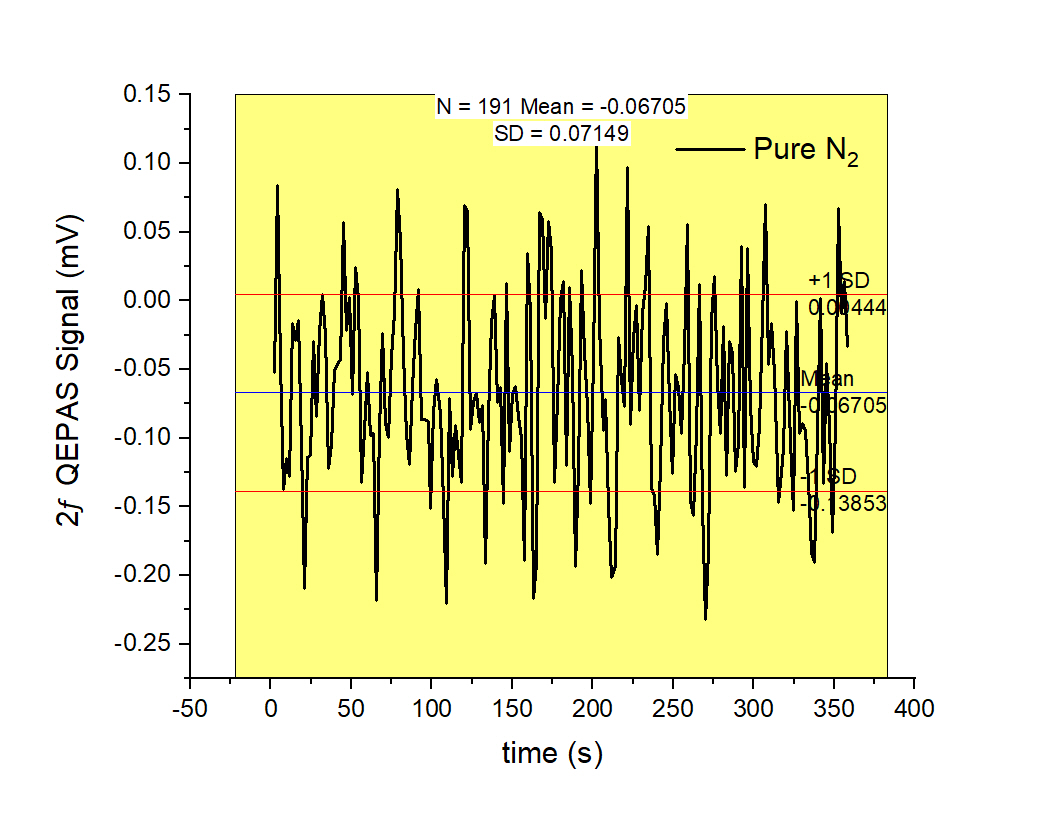

Supplement: MMC S3 — . [file mmc3.zip › Figure_S3_V2.jpg]
